# The mediating role of blood metabolites in the association between myocardial infarction and cancer risk: An observational and mendelian randomization analysis

**DOI:** 10.1371/journal.pone.0336980

**Published:** 2025-11-14

**Authors:** Jia Zhu, Xiaojun Xia, Haodong Jiang, Congying Wang, Yunpeng Jin

**Affiliations:** Department of Cardiology, Center for Cardiology, The Fourth Affiliated Hospital of School of Medicine, and International School of Medicine, International Institutes of Medicine, Zhejiang University, Yiwu, China; Shanghai Jiao Tong University, CHINA

## Abstract

**Background:**

Myocardial infarction (MI) and cancer are major global public health challenges. Research indicates that they share common risk factors and that physiological changes following MI may affect cancer incidence and progression. However, evidence defining the independent relationship between these conditions is still limited.

**Methods:**

We analyzed data from the National Health and Nutrition Examination Survey (NHANES) (2011–2018) using multivariable weighted logistic regression to examine the association between myocardial infarction (MI) and cancer. Additionally, we utilized genome-wide association study (GWAS) summary statistics and conducted Mendelian randomization (MR) to assess potential causal relationships and explore underlying mechanisms. Sensitivity analyses were performed to ensure the robustness of our findings.

**Results:**

A total of 20,859 participants were included in our observational study using NHANES data. Multivariable weighted logistic regression revealed no direct association between MI and cancer (OR=1.161, 95% CI [0.895–1.507], P = 0.261). Interestingly, MR analysis indicated that MI occurrence was associated with a reduced incidence of cancer (OR=0.9497, 95% CI [0.9223–0.9778], P = 0.0005). Furthermore, two-stage MR results suggested this reduction might be mediated by increased blood levels of metabolites that inhibit cancer development, such as dihomo-linoleate (20:2n6) (beta = −0.0050, 95% CI [−0.0027–0.0004], P < 0.0001), alpha-tocopherol (beta = −0.0042, 95% CI [−0.0060–0.0025], P < 0.0001), inosine (beta = −0.0015, 95% CI [−0.0027–0.0004], P = 0.0084), and methyl glucopyranoside (alpha + beta) levels (beta = −0.0006, 95% CI [−0.0010–0.0003], P = 0.0008).

**Conclusion:**

Our integrative analysis suggests that myocardial infarction may be associated with a reduced cancer incidence through potential alterations in blood metabolite profiles, including dihomo-γ-linolenic acid, alpha-tocopherol, and inosine. These findings provide preliminary evidence that warrants further large-scale studies to validate the observed associations and to elucidate the underlying mechanisms.

## Background

Myocardial infarction (MI) is the most severe clinical manifestation of coronary artery disease [1]. It poses a significant threat to human life and is one of the leading causes of death globally [2]. Furthermore, the occurrence of MI can lead to numerous long-term health issues, which is why MI continues to receive widespread attention from the medical community [3].

Cancer is similarly one of the leading causes of death worldwide [4]. According to the latest statistics, 2022 saw 20 million new cancer cases and 9.7 million cancer-related deaths, underscoring cancer as a persistent challenge to public health [5].

In summary, both diseases contribute significantly to the global health burden. However, whether these two conditions have an interrelated effect or independently contribute to the global disease burden remains a subject of debate. Some studies suggest that the occurrence of MI and caner share several risk factors, including smoking [6], obesity [7], and low physical activity [8]. There are multiple potential mechanisms that could link the occurrence of MI and cancer. For example, MI can induce chronic inflammation in the body [9], which may promote cancer development to some extent [10,11]. However, other research leans towards the view that MI is highly unlikely to cause new-onset cancer [12,13]. After controlling for comorbidities, the relationship between MI and cancer does not remain significant, and the higher cancer incidence post-MI may largely be attributable to increased routine medical checkups, leading to detection bias. Despite these mixed findings, studying myocardial infarction (MI) as a potential risk or protective factor for cancer remains important for several reasons. First, both diseases are major global health burdens and share numerous risk factors, including smoking, obesity, and physical inactivity, suggesting potential overlapping pathophysiology. Second, MI can trigger systemic biological changes—such as immune dysregulation, persistent low-grade inflammation, and metabolic reprogramming—that may either promote or inhibit tumorigenesis. Third, increasing evidence suggests that cardiovascular events, such as MI, can affect long-term outcomes in cancer patients, raising the question of whether similar mechanisms operate in the reverse direction. Finally, identifying whether MI contributes to cancer risk or protection can inform surveillance strategies and guide clinical decision-making in post-MI patients. Thus, clarifying this relationship has both mechanistic and public health relevance.

Metabolomics can reveal the relationship between metabolites or metabolic pathways and physiological or pathological changes, providing new insights into disease mechanisms [14]. Several studies have shown that the occurrence of MI induces alterations in certain metabolites [15], and there is also evidence suggesting that changes in metabolite levels can influence cancer development [16]. For example, alterations in lipid metabolism, amino acid pathways, and nucleotide synthesis have been shown to affect tumor growth, metastasis, and response to therapy [17,18].

Mendelian Randomization (MR) analysis uses genetic variants as instrumental variables (IVs) to assess causal relationships between exposure and outcomes, allowing better control for confounding factors. As a “naturally occurring randomized double-blind trial,” MR is a valuable complement to randomized controlled trials (RCTs). Given the inconsistent results from observational studies, MR research can serve as a reliable supplement [19]. Therefore, in this study, we first utilized a large-scale observational study from NHANES 2011–2018 to preliminarily explore the relationship between MI and cancer. This was followed by a two-sample Mendelian Randomization (MR) analysis to complement the findings. Finally, a two-step Mendelian Randomization (MR) analysis was employed to assess the mediating effect of metabolites in the exposure-outcome relationship.

## Methods

### Overall study design

The study is divided into two parts. In the first part, we utilized data from the National Health and Nutrition Examination Survey (NHANES) to investigate the relationship between myocardial infarction and cancer, adjusting for relevant confounders. In the second part, we used genetic data from a genome-wide association study (GWAS) through Mendelian randomization to explore the causal relationship between myocardial infarction and cancer and studied potential blood metabolites that might influence the relationship between the two.

### Ethics approval and consent to participate

The data utilized in this study were sourced from publicly available databases: FinnGen, UK Biobank, IEU Open GWAS Project, and NHANES. Each of these studies received approval from their respective ethical review committees and obtained written informed consent from the participants. Therefore, no additional ethics approval is required for this study.

#### Observational study.

***Study population in NHANES*:** The data used in the current analysis are publicly available through the NHANES database (https://www.cdc.gov/nchs/nhanes/index.htm). The protocols of the NHANES study were authorized by the Research Ethics Review Board of NCHS. Informed consent was obtained from all the NHANES participants. The study was exempt from the approval of the institutional review board as it used de-identified, publicly available data.

**Fig 1:**
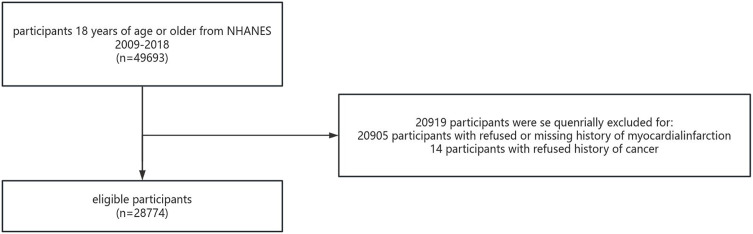
NHANES 2009-2018 participant screening process.

Participants over the age of 20 who attended the NHANES Mobile Examination Center from 2011–2018 were considered for inclusion in the study. After excluding those uncertain about their myocardial infarction and cancer status, the final cohort comprised 20,859 individuals. The specific recruitment process is illustrated (Fig 1).

***Definition and assessment of myocardial infarction and cancer in NHANES*:** Information regarding whether participants had suffered a myocardial infarction was obtained through a questionnaire. Those who answered ‘Yes’ to the question ‘Has a doctor or other health professional ever told you that you had a myocardial infarction?’ were considered to have a history of myocardial infarction. The definition of cancer adhered to the current NCI definition: ‘Cancer is a disease in which some of the body’s cells grow uncontrollably and spread to other parts of the body.’ Similarly, data on cancer history were also collected via questionnaire, with participants who reported a history of cancer being classified as having the disease.

***Other covariates used in NHANES*:** To maximize control of confounding factors, we adjusted for variables that have been identified in the literature as relevant to both myocardial infarction and cancer [13,20,21]. adjustments were made for the following variables: gender, age, race, education level, household income, waist circumference, BMI, LDL, HDL, triglycerides, smoking status, alcohol consumption, diabetes status, vigorous work activities, and moderate rest activities. All variables were considered potential confounders affecting the relationship between myocardial infarction and cancer. Smokers were categorized into three groups based on frequency: everyday, someday, and not at all. Alcohol consumption was classified according to frequency as well: Never, Every day, Nearly every day, 3–4 times a week, 2 times a week, Once a week, 2–3 times a month, Once a month, 7–11 times in the last year, 3–6 times in the last year, and 1–2 times in the last year. Diabetes was defined as a history of previous diabetes, an HbA1c level ≥6.5%, or a fasting blood glucose level ≥126 mg/dL. Vigorous work situations were mainly categorized based on whether participants engaged in high-intensity activities that significantly increase breathing or heart rate. Moderate recreational activities refer to participation in any medium-intensity exercises, fitness, or recreational activities that cause a slight increase in breathing or heart rate.

***Statistical analysis*:** In our analysis using the NHANES data, we employed multivariable logistic regression to assess the odds ratios (OR) and 95% confidence intervals (CI) for myocardial infarction and cancer. Three models were constructed:Model 1: Unadjusted. Model 2: Adjusted for age (continuous), sex (Male, Female), and race (Mexican American, Other Hispanic, Non-Hispanic White, Non-Hispanic Black, Other Race – Including Multi-Racial). Model 3: Further adjusted for education level (Less than 9th grade, 9–11th grade, High school, Some college or AA degree, College graduate or above), a ratio of family income to poverty guidelines (continuous), BMI (kg/m^2^), waist circumference (cm), HDL-cholesterol (mg/dL), LDL-cholesterol (mg/dL), and triglycerides (mg/dL). Any individuals with missing covariate data were excluded from the analyses in Model 2 and Model 3. All the analyses were performed with R (version 4.3.3. http://www.R-project.org) and EmpowerStats software (http://www.empowerstats.com). The value of p < 0.05 was considered statistically significant.

#### Mendelian randomization.

***Study design*:** In this study, a two-sample Mendelian Randomization (MR) framework is employed to rigorously evaluate the causal relationship between myocardial infarction and cancer, while also using a two-step Mendelian method to study the blood metabolites that potentially influence this relationship. The mediation proportions were calculated using the formula: (β1 × β2)/β, where β represents the total effect derived from the primary analysis, β1 represents the effect of myocardial infarction traits on the mediators, and β2 represents the effect of the mediators on cancer. Standard errors and confidence intervals (CIs) were calculated using the delta method [22]. In particular, the Single Nucleotide Polymorphisms (SNPs) used as Instrumental Variables (IVs) in the MR analysis must fulfill three stringent criteria: 1) They must have a significant association with the exposure; 2) They must be independent of the outcome variable 3) They must not be related to any confounding variables. This research adheres to the STROBE-MR guidelines [23].

***Genome-wide association study (GWAS) data sources for myocardial infarction, cancer and metabolites*:** Genetic variations related to myocardial infarction were derived from Genome-wide analysis identifies novel susceptibility loci for myocardial infarction (N_case_ = 61505, N_control_ = 577720), with the associated accession numbers GCST011365. This meta-analysis included data from the UK Biobank (n ≈ 472,000) and the CARDIoGRAMplusC4D Consortium (n ≈ 167,000) [24]. We obtained overall cancer data from the FinnGen database, including data on various cancer subtypes categorized by anatomical site: head and neck, respiratory intrathoracic, digestive organs, bone cartilage, skin, mesothelioma soft tissue, breast, urinary tract, bladder, eye brain neuro, and endocrine. FinnGen, a large-scale database, provided the GWAS data on cancer, encompassing 314,193 participants, with cancer case numbers ranging from 64 to 87,531. All data can be accessed at https://www.finngen.fi/en/access_results. Summary statistics of plasma metabolomics were also obtained from the GWAS Catalog. This study analyzed data from 8,299 European individuals, encompassing 1,091 plasma metabolites and 309 metabolite ratios, with the associated accession numbers GCST90199621–GCST90201020. Among the 1,091 plasma metabolites, 850 were identified, and these could be categorized into eight major metabolic groups: lipids (395), amino acids (210), xenobiotics (130), nucleotides [25], cofactors and vitamins [26], carbohydrates [20], peptides [15], and energy-related metabolites [8]. The remaining metabolites included partially characterized molecules [15] and unidentified compounds (220) [27] (S1 Table).

***Selection of instrumental variables*:** To meet the assumptions mentioned above, we conducted a stringent selection of SNPs. Each SNP had to exhibit significant genome-wide association (P-value < 5 × 10^-8) [28]. All SNPs used as instrumental variables (IVs) were processed using PLINK software (version v1.90), meeting the linkage disequilibrium criterion (r^2 < 0.001, 10,000 kb) based on the 1000 Genomes Project Phase 3 LD data [29]. Subsequently, we systematically excluded IVs with F-statistics < 10 [30]. Finally, we utilized the Phenoscanner database (http://www.phenoscanner.medschl.cam.ac.uk/) to eliminate confounding factors related to the outcomes [26,31] and replicated the above results using R programming language (S1 File).

***Statistical analysis*:** All analyses were rigorously evaluated using the “Mendelian-Randomization” package (version 0.4.3). We employed the Inverse Variance Weighted (IVW) method as the primary approach to assess the causal relationship between myocardial infarction (MI) and cancer, as well as the mediating effects of blood metabolites [25,32]. Additionally, we supplemented our findings with four methods: MR Egger, Weighted Median, Simple Mode, and Weighted Mode [33]. To enhance the reliability of our results, we calculated Cochran’s Q statistic and its corresponding P-value to comprehensively quantify heterogeneity among the instrumental variables (IVs) used [34]. Furthermore, we utilized the intercept of MR-Egger to evaluate horizontal pleiotropy [35]. The leave-one-out analysis was conducted to assess the potential influence of any single SNP on the overall association [36].

To further explore the mediating role of blood metabolites in the relationship between myocardial infarction and cancer, we implemented a two-step Mendelian Randomization (MR) analysis framework. In the first step, genetic variants associated with myocardial infarction were used as instrumental variables to estimate their effects on circulating metabolite levels (exposure → mediator). In the second step, the same genetic variants were used to evaluate the causal effects of the identified metabolites on cancer risk (mediator → outcome). This approach allows us to estimate the indirect (mediated) effect via each metabolite, in addition to the total and direct effects. Mediation proportion was calculated using the product of coefficients method:


$$mediation\;effe\text{ct}=\sqrt{\left(\beta_{\text{1}}^{\text{2}})(se_{\text{1}}^{\text{2}})+(\beta_{\text{2}}^{\text{2}})(se_{\text{2}}^{\text{2}}\right)}$$

where *β*_1_ is the effect of MI on the metabolite, and *β*_2_ is the effect of the metabolite on cancer. Standard errors and confidence intervals were derived using the delta method. This two-step MR approach helps to identify potential metabolic pathways that mediate the protective effects of MI on cancer risk.

To assess the robustness and reliability of our two-sample Mendelian Randomization (2SMR) analyses, we evaluated the statistical power using the mRnd web tool (https://shiny.cnsgenomics.com/mRnd/). For the primary analysis assessing the causal effect of myocardial infarction on cancer, assuming an R^2^ of 0.0064 for the genetic instruments (based on GWAS data), a sample size of approximately 639225 (40 SNPs), and a type I error rate of 0.05, the power to detect an odds ratio (OR) of 0.95 or lower was estimated to be > 90%. For the reverse-direction analysis (cancer → MI), with similar instrument strength but smaller case sample sizes for certain cancer subtypes, the power was notably lower, particularly for associations with weaker effect sizes (OR > 0.95). These results suggest our primary 2SMR analyses are adequately powered, while some reverse MR analyses should be interpreted with caution due to limited statistical power.

## Results

### General characteristics of NHANES

In this study, a total of 28,774 participants were included, comprising 1,213 individuals with myocardial infarction and 27,561 without. Compared to those without myocardial infarction, participants with myocardial infarction were more likely to be male, older, have a higher body mass index (BMI), larger waist circumference, and lower levels of high-density lipoprotein (HDL). Additionally, they had a lower level of education, engaged in less healthy lifestyle behaviors, and had a higher incidence of cancer (Table 1).

**Table 1:** Demographic and clinical characteristics of participants with and without myocardial infarction.

Myocardial infarction	No	Yes	*P*-value
	N = 27561	N = 1213	
Age,(years)	48.964 ± 17.644	66.766 ± 11.881	<0.001
Gender,%			<0.001
Male	13122 (47.611%)	801 (66.035%)	
Female	14439 (52.389%)	412 (33.965%)	
Race,%			<0.001
Mexican American	4039 (14.655%)	123 (10.140%)	
Other Hispanic	2891 (10.489%)	104 (8.574%)	
Non-Hispanic White	10612 (38.504%)	652 (53.751%)	
Non-Hispanic Black	6018 (21.835%)	228 (18.796%)	
Other Race – Including Multi-Racial	4001 (14.517%)	106 (8.739%)	
Education level,%			<0.001
Less than 9th grade	2741 (9.945%)	189 (15.581%)	
9–11th grade	3677 (13.341%)	206 (16.983%)	
High school	6137 (22.267%)	309 (25.474%)	
Some college or AA degree	8309 (30.148%)	315 (25.969%)	
College graduate or above	6660 (24.165%)	191 (15.746%)	
A ratio of family income to poverty guidelines	2.484 ± 1.634	2.067 ± 1.471	<0.001
BMI,(kg/m²)	29.240 ± 7.071	30.264 ± 7.198	<0.001
Waist Circumference,(cm)	99.212 ± 16.471	106.502 ± 16.044	<0.001
HDL-Cholesterol,(mg/dL)	53.272 ± 16.203	48.666 ± 15.600	<0.001
LDL-Cholesterol, (mg/dL)	113.507 ± 35.291	96.378 ± 37.045	<0.001
Triglyceride,(mg/dL)	121.760 ± 108.621	131.522 ± 86.938	0.039
Smoking status,%			0.066
Every day	1042 (38.808%)	56 (32.749%)	
Some days	233 (8.678%)	10 (5.848%)	
Not at all	1410 (52.514%)	105 (61.404%)	
Drinking status,%			<0.001
Never	943 (22.739%)	88 (40.183%)	
Every day	131 (3.159%)	10 (4.566%)	
Nearly every day	145 (3.497%)	9 (4.110%)	
3–4 times a week	262 (6.318%)	7 (3.196%)	
2 times a week	300 (7.234%)	17 (7.763%)	
Once a week	314 (7.572%)	17 (7.763%)	
2–3 times a month	561 (13.528%)	7 (3.196%)	
Once a month	318 (7.668%)	15 (6.849%)	
7–11 times in the last year	273 (6.583%)	8 (3.653%)	
3–6 times in the last year	427 (10.297%)	16 (7.306%)	
1–2 times in the last year	469 (11.309%)	25 (11.416%)	
Diabetes,%			<0.001
No	23461 (87.313%)	726 (61.893%)	
Yes	3409 (12.687%)	447 (38.107%)	
Vigorous work activity,%			0.001
No	22244 (80.735%)	1024 (84.419%)	
Yes	5308 (19.265%)	189 (15.581%)	
Moderate recreational activities,%			<0.001
No	16376 (59.424%)	858 (70.734%)	
Yes	11182 (40.576%)	355 (29.266%)	
Cancer,%			<0.001
Cancer	2518(9.136%)	269 (22.176%)	
No	25043 (90.864%)	944 (77.906%)	

Note: A total of 28,774 participants were included in this analysis (1,213 with myocardial infarction, 27,561 without).

### Logistic regression between myocardial infarction and cancer

We employed logistic regression models to investigate the relationship between myocardial infarction and cancer (Table 2). In both Model 1 and Model 2, our findings indicated that the occurrence of myocardial infarction was associated with an increased risk of developing cancer. (OR=2.834, 95%CI [2.46,3.265], P < 0.001; OR=1.204, 95%CI [1.034,1.403], P = 0.0169)However, after adjusting for all covariates, the relationship between myocardial infarction and cancer became less robust (OR=1.161, 95%CI [0.895–1.507], P < 0.261).

**Table 2:** Association between myocardial infarction and cancer.

Myocardial infarction status	Model 1 or (95% CI) *P*-value	Model 1 or (95% CI) *P*-value	Model 1 or (95% CI) *P*-value
No	1.0 (reference)	1.0 (reference)	1.0 (reference)
Yes	2.834 (2.460, 3.265) <0.001	1.204 (1.034, 1.403) 0.017	1.161 (0.895,1.507) 0.261
Model 1: unadjusted model. Model 2: adjusted for age (continuous), sex (Male, Female) and race (Mexican American, Other Hispanic, Non-Hispanic White, Non-Hispanic Black, Other Race – Including Multi-Racia). Model 3: adjusted for age (continuous), sex (Male, Female), race (Mexican American, Other Hispanic, Non-Hispanic White, Non-Hispanic Black, Other Race – Including Multi-Racia), education level (Less than 9th grade, 9–11th grade, High school, Some college or AA degree, College graduate or above), A ratio of family income to poverty guidelines (continuous), BMI (kg/m^2^), Waist Circumference (cm), HDL-Cholesterol (mg/dL), LDL-Cholesterol (mg/dL), Triglyceride (mg/dL), lifestyle behaviors, diabetes.			

Note: Logistic regression models were conducted on 20,859 participants with complete data for all covariates.

### Causal effect from myocardial infarction to cancer

**Fig 2:**
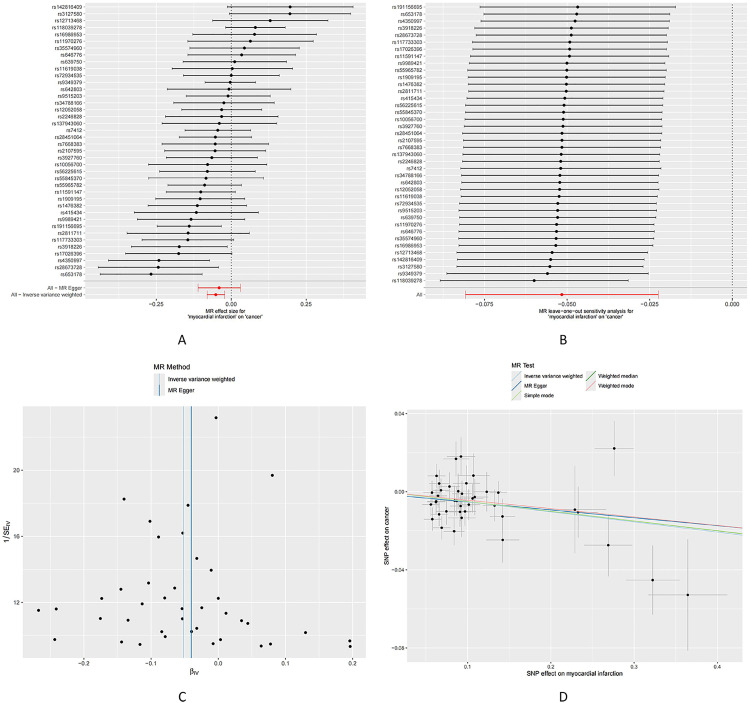
Causal effects of myocardial infarction on cancer. A: Forest plot summarizing myocardial infarction’s overall impact on cancer. B: Sensitivity analysis via “leave-one-out” plots. C: Funnel plot for bias assessment of the estimates. D: Scatter plot of myocardial infarction effect estimates on cancer.

Mendelian randomization analysis results indicate that myocardial infarction (MI) exhibits a protective effect on both overall cancer risk and several cancer subtypes. Specifically, protective associations were observed for basal cell carcinoma (OR=0.9268, 95%CI [0.8839, 0.9717], P = 0.0016), bronchus and lung cancer (OR=0.8142, 95%CI [0.7473, 0.8871], P < 0.001), colon cancer (OR=0.8749, 95%CI [0.7818, 0.9790], P = 0.0198), colorectal cancer (OR=0.8991, 95%CI [0.8140, 0.9931], P = 0.0361), head and neck cancer (OR=0.8459, 95%CI [0.7497, 0.9545], P = 0.0066), kidney (excluding renal pelvis) cancer (OR=0.8763, 95%CI [0.7720, 0.9946], P = 0.0409), pancreatic cancer (OR=0.7968, 95%CI [0.6923, 0.9171], P = 0.0015), skin cancer (OR=0.9351, 95%CI [0.8927, 0.9796], P = 0.0046), and tonsil base tongue cancer (OR=0.7115, 95%CI [0.5431, 0.9322], P = 0.0135). Additionally, Cochrane’s Q test did not detect heterogeneity, and the MR-Egger intercept showed no evidence of directional pleiotropy concerning myocardial infarction. For other cancer subtypes, the Mendelian randomization analysis did not reveal a clear causal relationship (Table 3; Fig 2; S2 and S3 Tables).

**Table 3:** MR results for the relationship between myocardial infarction on cancer.

Exposure	Outcome	No. of SNP	Methods	OR (95%CI)	P	Heterogeneity test		Pleiotropy test
						Cochran’s Q (I^2^)	P	p pintercept
Myocardial infarction	Cancer	40	MR Egger	0.9605 (0.8949-1.031)	0.2710	55.9473 (32.08%)	0.0303	0.7323
		40	Weighted median	0.9512 (0.9159-0.9880)	0.0098			
		40	IVW	0.9497 (0.9224-0.9778)	0.0005	56.1221 (30.57%)	0.0372	
		40	Simple mode	0.9515 (0.8827-1.0257)	0.2023			
		40	Weighted mode	0.9579 (0.8930-1.0276)	0.2373			

Note: Summary statistics were derived from GWAS datasets with sample sizes ranging from approximately 300,000–600,000 individuals depending on the trait and cancer subtype (detailed in Methods and S2–S6 Tables).

### Causal effect from cancer to myocardial infarction

**Fig 3:**
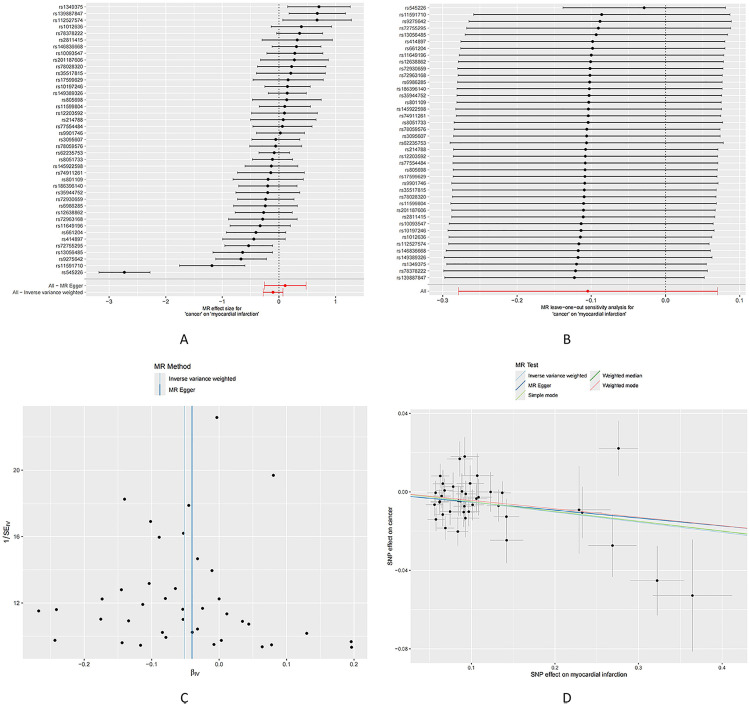
Causal effects of cancer on myocardial infarction. A: Forest plot summarizing cancer’s overall impact on cancer. B: Sensitivity analysis via “leave-one-out” plots. C: Funnel plot for bias assessment of the estimates. D: Scatter plot of effect estimates cancer on myocardial infarction.

In the reverse-direction Mendelian randomization analysis, we observed significant associations with several cancer types, including colon cancer (OR=0.9628, 95% CI [0.9284, 0.9986], P = 0.0416), fibrosarcoma (OR=0.9870, 95% CI [0.9755, 0.9987], P = 0.0296), hepatocellular carcinoma (OR=0.9870, 95% CI [0.9755, 0.9987], P = 0.0017), and mantle cell lymphoma (OR=0.9815, 95% CI [0.9698, 0.9933], P = 0.0022). However, no statistically significant associations were observed for other cancer subtypes or the overall cancer risk (OR=0.9007, 95% CI [0.7565, 1.0724], P = 0.2402) (Table 4; Fig 3; S4 and S5 Tables).

**Table 4:** MR results for the relationship between cancer on myocardial infarction.

Exposures	Outcomes	No. of SNP	Methods	OR (95%CI)	P	Heterogeneity test		Pleiotropy test
						Cochran’s Q (I^2^)	P	p pintercept
Cancer	Myocardial infarction	41	MR Egger	1.1156 (0.7725-1.6111)	0.5630	204.9560 (80.97%)	<0.0001	0.2035
		41	Weighted median	0.9443 (0.8362-1.0664)	0.3558			
		41	IVW	0.9007 (0.7565-1.0724)	0.2402	213.7448 (81.29%)	<0.0001	
		41	Simple mode	1.1155 (0.8846-1.4068)	0.3612			
		41	Weighted mode	0.9599 (0.7924-1.1630)	0.6786			

### Mediation analyses of potential blood metabolites

To explore the potential mechanisms through which myocardial infarction may reduce the incidence of cancer, we utilized genetic data and employed a two-step Mendelian randomization (MR) approach to examine the relationships between 1,091 plasma metabolites and 309 metabolite ratios with myocardial infarction and cancer. To address the potential inflation of type I error due to multiple testing, we applied a false discovery rate (FDR) corrected significance threshold of P < 0.05. The results showed that only 3 plasma metabolites had significant negative mediation effects on the relationship between myocardial infarction and cancer, Dihomo-linoleate (20:2n6) (beta = −0.0050, 95%CI [−0.0027,-0.0004], P = 0.0042), Alpha-tocopherol (beta = −0.0042, 95%CI [−0.0060,-0.0025], P < 0.0001), Methyl glucopyranoside (alpha + beta) levels (beta = −0.0006, 95%CI [−0.0010, −0.0003], P = 0.0184). Additionally, we also found that the ratio change of one metabolite showed similar mediation relationship, Arginine to ornithine ratio (beta = −0.0018, 95%CI [−0.0027, −0.0009], P = 0.0031) (Table 5, S6 Table).

**Table 5:** Mediation effect of myocardial infarction on cancer via blood metabolites.

Exposure	Mediator	Outcome	Total effect	Direct effect	Mediation effect (95% CI)	P	Mediation Proportion (95% CI)
Myocardial infarction	Dihomo-linoleate (20:2n6) levels	Cancer	−0.0516	−0.0466	−0.0050 (−0.0074, −0.0026)	0.0042	9.65% (4.98%, 14.30%)
	Alpha-tocopherol levels			−0.0474	−0.0042 (−0.0060, −0.0025)	<0.0001	8.15% (4.75%, 11.6%)
	Methyl glucopyranoside (alpha + beta) levels			−0.0510	−0.0006 (−0.0010, −0.0003)	0.0184	1.24% (0.52%, 1.97%)
	Arginine to ornithine ratio			−0.0498	−0.0018 (−0.0027, −0.0009)	0.0031	3.48% (1.83%, 5.13%)

These metabolites were identified through an unbiased, hypothesis-free screening of 1,091 plasma metabolites and 309 metabolite ratios using two-step Mendelian Randomization (MR) analysis. Only those metabolites showing statistically significant mediation effects (P < 0.05, further corrected via Bonferroni adjustment) were reported. The selection was therefore driven by statistical evidence of both (1) MI influencing the metabolite, and (2) the metabolite influencing cancer.

In terms of biological plausibility, several of the identified metabolites have well-documented roles in modulating inflammatory or oxidative stress pathways associated with carcinogenesis. For example, dihomo-linoleate (20:2n6) is a long-chain polyunsaturated fatty acid that can modulate membrane signaling and lipid peroxidation. Alpha-tocopherol (vitamin E) is an antioxidant known to suppress free radical formation and tumor growth. Inosine, a purine nucleoside, has been implicated in enhancing T cell-mediated antitumor immunity and modulating RNA metabolism. These biological roles are supported by prior experimental and epidemiological studies, as cited in our Discussion section. While methyl glucopyranoside and arginine to ornithine ratio are less well-characterized in cancer biology, they may reflect broader alterations in glucose and amino acid metabolism that are frequently observed in both cardiovascular and oncological contexts.

While statistical mediation proportions for these metabolites were generally small (<10%), the consistency across MR methods, combined with known biological functions of several of the metabolites, increases our confidence in their potential mediating roles. Nevertheless, we acknowledge that these findings are exploratory in nature and require validation in experimental or longitudinal cohorts.

## Discussion

To the best of our knowledge, this is the first study to explore the relationship between myocardial infarction and cancer using both large-scale observational data and genetic data through MR analysis. Our observational studies initially suggested an increased incidence of cancer among patients with myocardial infarctions, but this association was not robust after adjusting for additional factors. In our exploratory two-sample MR analysis, we observed genetic evidence suggesting a potential inverse association between genetic liability to MI and the risk of several site-specific cancers. This study is among the first to generate the hypothesis that a genetic predisposition to myocardial infarction might be associated with a lower risk of certain cancers, particularly for basal cell carcinoma, bronchus and lung cancer, colon cancer, colorectal cancer, head and neck cancer, kidney cancer (excluding renal pelvis), pancreatic cancer, skin cancer, and tonsil base tongue cancer. Cohort studies examining the relationship between myocardial infarction and cancer are currently scarce and yielding inconsistent conclusions [12,37]. L Dreyer et al. [38] found no significant genetic predisposition to cancer following myocardial infarction after excluding smoking—a common risk factor. This finding aligns with our observational study results. Another research performed by Maarten J G Leening et al. [13] suggests that early stages of myocardial infarction could promote the onset of cancer, though this association dissipates over time. They hypothesized that myocardial infarction might trigger an unrecognized paraneoplastic syndrome, facilitating cancer through mechanisms promoting inflammation and anemia [20]. Frequent blood testing could also increase the detection rates of hematologic cancers [37,39]. It is important to note that although the cancer data in studies by Maarten J G Leening et al. primarily come from cancer registries, these studies might be biased due to increased routine medical examinations following myocardial infarction, a type of bias reported in other studies. Additionally, Morten Malmborg et al [40]. noted that despite an initial promotion of cancer by myocardial infarction, there was no significant increase in cancer incidence within the first six months post-infarction after adjusting for factors such as chronic obstructive pulmonary disease, hypertension, dyslipidemia, diabetes, and socioeconomic status.

MR studies, based on genetic variants, show significant advantages in minimizing selection biases compared to traditional observational studies [41]. Additionally, MR provides unique benefits in assessing long-term health effects compared to randomized controlled trials [42,43]. To ensure the validity of our MR findings, we conducted multiple statistical tests, including the MR-Egger intercept test to rule out pleiotropy, Cochrane’s Q test, and leave-one-out sensitivity analyses to confirm the robustness of our findings. Furthermore, bidirectional MR analyses helped eliminate potential reverse causality [44].

To investigate the potential mechanisms by which myocardial infarction may lead to a reduction in cancer occurrence, we focused on identifying blood metabolites. We calculated their mediation proportion using a two-stage MR method. Additionally, several existing studies have provided plausible biological pathways that could support this hypothesis. For instance, Xiaoping Wang et al. discovered that dihomo-γ-linolenic acid can be further metabolized by inflammatory cells into 15-(S)-hydroxy-8,11,13-eicosatrienoic acid and prostaglandin E1 (PGE1), which subsequently inhibit cancer proliferation [17]. A longitudinal cohort study confirmed that α-tocopherol can eliminate free radicals, inhibit carcinogen formation and tumor growth, and stimulate cancer cell apoptosis [18]. Moreover, inosine has been shown to regulate RNA translation and stability, thereby modulating cancer cell gene expression. It significantly enhances T cells’ ability to attack cancer cells, improving the efficacy of immune checkpoint inhibitors, such as PD-1 antibodies [45–47]. The protective association between myocardial infarction and cancer observed in the MR analysis is indeed counterintuitive and diverges from the initially positive association observed in the unadjusted observational models. Several factors may explain this discrepancy. First, MR analysis estimates lifetime exposure effects based on genetic predisposition, whereas observational studies reflect post-event biological changes and clinical behaviors. Thus, MR may capture the long-term systemic effects of genetically predicted myocardial infarction risk, independent of acute clinical events or their sequelae. Second, MR analysis is less prone to confounding and reverse causality, which may obscure the directionality of associations in NHANES data. Third, the possibility of horizontal pleiotropy or weak instrument bias was rigorously evaluated using MR-Egger intercepts, heterogeneity statistics, and leave-one-out sensitivity analyses. No significant evidence of pleiotropy was found, and the effect estimates remained robust across multiple MR methods (IVW, weighted median, MR Egger), supporting the reliability of the findings. Nonetheless, we recognize that the overall effect size was modest (OR=0.949), and further replication in independent cohorts is warranted. Finally, the discordance between MR and observational estimates may reflect differences in temporal dynamics, sample composition, and the potential detection bias in clinical settings following myocardial infarction.

A notable strength of this study is the use of the extensive NHANES database, which includes a large sample size, lending representativeness to our observational findings. Moreover, by integrating observational and MR methods, we comprehensively explored the relationship between myocardial infarction and cancer and investigated the potential mechanisms underlying this relationship. The extensive overlap in risk factors and disease prevention for cardiovascular disease and cancer suggests that these seemingly diverse diseases have some common basic molecular pathways ornetworks [13,37,48]. In other words, controlling risk factors for myocardial infarction can help reduce the risk of cancer. As demonstrated in our study, better control of risk factors after myocardial infarction may help lower the cancer risk in these patients. Additionally, myocardial infarction induces changes in blood metabolites, which can, to some extent, inhibit cancer occurrence. Further understanding of the intricate interactions between myocardial infarction and cancer may lead to better prevention, earlier detection, and safer treatment strategies.

It is also important to acknowledge potential biases that may affect the observed association between myocardial infarction and cancer. First, the null association observed in the fully adjusted observational model (P = 0.261) may be influenced by competing risks, particularly in older individuals. Patients with myocardial infarction may die from cardiovascular causes before a cancer diagnosis can be made, thereby artificially lowering the observed cancer incidence in this population. A competing risk model, such as Fine and Gray’s subdistribution hazard model, may be more appropriate for estimating cancer risk in the presence of high cardiovascular mortality. Second, individuals with a history of MI often undergo more frequent and intensive health surveillance, which could increase the likelihood of incidental cancer detection, especially in the early stages. This surveillance bias might confound the observed associations, leading to overestimation of cancer risk in myocardial infarction patients. Future studies incorporating competing risk modeling and time-dependent surveillance metrics are warranted to further clarify these effects.

However, the limitations of our study should not be overlooked. First, the effect size observed in our final results is relatively small (OR=0.949), which may be attributed to several factors. Despite controlling for various confounders, the multifactorial nature of both myocardial infarction and cancer could still be influenced by unmeasured genetic and environmental factors. On the other hand, as the analysis is based on the overall effect of myocardial infarction on cancer, it is evident that MI shows a stronger association with certain cancer types, while the relationship with most other cancer types is weaker. This contributes to the small overall odds ratio (OR) observed. Moreover, our MR analysis only adjusted for a subset of confounders, such as smoking and alcohol consumption, and may not have fully accounted for all potential confounding variables. Additionally, the genetic data used in MR analysis inherently contains variability and noise, which could have influenced the results. The exclusion of Asian populations may limit the generalizability of our findings [49]. Finally, while MR analysis provides valuable statistical associations, further research is needed to elucidate the underlying biological mechanisms linking myocardial infarction and cancer.

Our findings, while novel, should be interpreted in the context of existing literature and the inherent limitations of the MR design. Several previous studies have reported conflicting associations between myocardial infarction and cancer incidence. For instance, Leening et al. (2023) reported an increased risk of cancer following ST-segment elevation myocardial infarction in older individuals, which they attributed to early inflammatory responses or possible paraneoplastic phenomena. In contrast, Dreyer and Olsen (2005) found no significant long-term association after adjusting for smoking and surveillance bias. More recently, Malmborg et al. (2023) emphasized that any apparent increase in cancer incidence following myocardial infarction was limited to the first six months and likely influenced by heightened medical attention. These discrepancies underscore the complexity of the MI–cancer relationship and the influence of study design, population characteristics, and follow-up duration. In this context, our MR-based approach provides a complementary perspective by suggesting that genetic liability to myocardial infarction might be associated with a reduced cancer risk, potentially through metabolic reprogramming. However, it is crucial to explicitly emphasize the exploratory nature of this protective effect. While MR reduces confounding and reverse causality, it relies on several key assumptions that are difficult to fully verify. Therefore, the observed inverse association should be considered primarily as hypothesis-generating. The modest effect size (OR=0.949), the possibility of residual pleiotropy despite our sensitivity analyses, and the inherent variability in genetic data all underscore the preliminary nature of this evidence. Consequently, our results do not establish a definitive protective effect of MI on cancer but rather highlight a novel genetic correlation that merits further investigation. Further mechanistic studies and prospective cohort validation in independent populations are needed to confirm this association and reconcile the divergent observations across studies.

## Conclusion

Our integrative analysis, combining observational and exploratory Mendelian randomization approaches, suggests that genetic liability to myocardial infarction might be linked to a reduced risk of certain cancers, potentially mediated by alterations in blood metabolites such as dihomo-γ-linolenic acid, alpha-tocopherol, and inosine. However, the evidence for this protective relationship is preliminary and derived from genetic instrumental variable analysis. Further large-scale studies, including prospective cohorts and experimental research, are imperative to validate this finding and elucidate the precise biological mechanisms involved.

## Supporting information

S1 Table Phenotype source and description. (XLS)

S2 Table The instrumental variables (IVs) used to analyze the relationship between myocardial infarction and cancer. (CSV)

S3 Table MR results for the relationship between myocardial infarction and various cancer. (XLS)

S4 Table The instrumental variables (IVs) used to analyze the relationship between cancer and myocardial infarction. (CSV)

S5 Table MR results for the relationship between various cancer and myocardial infarction. (XLS)

S6 Table Mediation effect of myocardial infarction on cancer via blood metabolites. (CSV)

S7 Table Pleiotropic effects of metabolites on cancer. (CSV)

S8 Table Pleiotropic effects of myocardial infarction on metabolites. (CSV)

S1 File The R code for Mendelian randomization analysis to remove confounding factors. (DOC)

## Abbreviations

IV: Instrumental variable
IVW: Inverse variance-weighted
MR: Mendelian randomization
MR-PRESSO: Mendelian Randomization Pleiotropy RESidual Sum and Outlier
NHANSE: National Health and Nutrition Examination Survey
MI: Myocardial Infarction.
