# Supplementary material for: The mediating role of blood metabolites in the association between myocardial infarction and cancer risk: An observational and mendelian randomization analysis
Source: PLoS One. 2025 Nov 14;20(11):e0336980. doi: 10.1371/journal.pone.0336980 (PMC12617889; doi:10.1371/journal.pone.0336980)
Supplement: S1 File — (DOC) [file pone.0336980.s009.doc]

FastDownloader::install_pkg("FastTraitR")

library(FastTraitR)

library(readr)

# Set the variable infile to store the file path of the exposure data.

infile <- "F06.csv"

# Read the exposure data file and store it in the variable data.

data <- read.csv(infile)

res = look_trait(file_name=infile, pval=1e-5,out_file = "confounder07.csv")

# Set the variable remove_snps to store the SNPs associated with confounding factors to be removed.

remove_snps <- c("", "","")

# Remove the rows containing SNPs associated with confounding factors.

data <- data[!data$SNP %in% remove_snps,]

# Write the results, after removing the confounding factors, to a file named clean_confounder07.csv.

write_csv(data, "clean_confounder07.csv")
